# Attitude and concerns of healthy individuals regarding post-mortem brain donation. A qualitative study on a nation-wide sample in Italy

**DOI:** 10.1186/s12910-023-00980-3

**Published:** 2023-11-27

**Authors:** Chiara Cattaneo, Iuliia Urakcheeva, Gianmarco Giacomini, Maria Antonietta Stazi, Susanna Lana, Antonio Arnofi, Miriam Salemi, Virgilia Toccaceli

**Affiliations:** 1 National Centre for Disease Prevention and Health Promotion, Istituto Superiore di Sanità, Rome, Italy; 2 Presidency of Istituto Superiore di Sanità, Rome, Italy; 3 Centre of Reference for Mental Health and Behavioural Sciences, Istituto Superiore di Sanità, Rome, Italy; 4 Department of Public Health Sciences, University of Turin, Turin, Italy

**Keywords:** Brain, Healthy individuals, Posthumous donation, Decision making, Informed consent

## Abstract

**Background:**

Collecting post-mortem brain tissue is essential, especially from healthy “control” individuals, to advance knowledge on increasingly common neurological and mental disorders. Yet, healthy individuals, on which this study is focused, are still understudied. The aim of the study was to explore, among healthy potential brain donors and/or donors’ relatives, attitude, concerns and opinion about post-mortem brain donation (PMBD).

**Methods:**

A convenience sampling of the general population (twins and their non-twin contacts) was adopted. From June 2018 to February 2019, 12 focus groups were conducted in four Italian cities: Milan, Turin, Rome and Naples, stratified according to twin and non-twin status. A qualitative content analysis was performed with both deductive and inductive approaches. Emotional interactions analysis corroborated results.

**Results:**

One hundred and three individuals (49–91 yrs of age) participated. Female were 60%. Participants had scarse knowledge regarding PMBD. Factors affecting attitude towards donation were: concerns, emotions, and misconceptions about donation and research. Religion, spirituality and secular attitude were implied, as well as trust towards research and medical institutions and a high degree of uncertainty about brain death ascertainment. Family had a very multifaceted central role in decision making. A previous experience with neurodegenerative diseases seems among factors able to favour brain donation.

**Conclusions:**

The study sheds light on healthy individuals’ attitudes about PMBD. Brain had a special significance for participants, and the ascertainment of brain death was a source of debate and doubt. Our findings emphasise the importance of targeted communication and thorough information to promote this kind of donation, within an ethical framework of conduct. Trust in research and health professionals emerged as an essential factor for a collaborative attitude towards donation and informed decision making in PMBD.

**Supplementary Information:**

The online version contains supplementary material available at 10.1186/s12910-023-00980-3.

## Background

Brain collections play an important role in advancing knowledge about neurological diseases and mental disorders [1]. Yet, it is still uncertain how to optimize individuals’ participation in brain tissue biobanking [2], taking into account the requirements of an ethical conduct concerning organs’ procurement and research with humans [3]. Indeed, recruitment of donors has been hindered by a decrease in post-mortem examinations, for many reasons [1, 4–7]. The need for control tissues to support neuroscience advancements is noticeable [8, 9], but recruiting “controls” is more problematic than recruiting patient donors: families of deceased patients affected by neurodegenerative disorders are more likely to consent to donation, or wishful to meet the desire of their deceased relative to donate his/her brain [10–13]. There are very few programmes focused on obtaining healthy control brains, largely based on medico-legal autopsies as a source of brain tissues, often without full clinical history and cognitive assessment data [1]. While obtaining control tissues for neuroscience remains a challenge, initiatives such as the Abbiategrasso Brain Bank Protocol are noteworthy. In fact, this is, so far, the first Italian cohort study involving brain donation, including both diseased and healthy donors, with the richness of data coming from longitudinal follow-ups from multiple perspectives (clinical, lifestyle, and biological samples collections) [14]. However, as the need for brain tissue increases, brain donation programmes and well-focused campaign strategies will be soon required to overcome the well-known shortage of high-quality brain tissue biospecimens and support scientific advances in increasingly common neurological disorders [15].

There is still a lot to understand about the attitude and concerns of healthy individuals towards post-mortem brain donation (PMBD). Being aware of motivations and cultural beliefs favouring or impeding the donation will be fundamental, as they might be both control donors and potential decision makers in the role of a deceased person’s close relative.

Those who consent to PMBD seem to be mostly influenced by the context and by the cause of death [16–18], but many other factors are likely to affect awareness of and attitude to PMBD. Most studies focused on few countries’ experience, e.g. Australia [19, 20] and UK [21]; they had heterogeneous approaches and targeted mostly patient-donor populations only, often precluding solid trasnferrable conclusions. Instead, studies regarding healthy population are quite dated [18, 19, 22, 23].

It is noteworthy that a great number of articles on brain bank construction were recently published, possibly reflecting an increased interest in this area of investigation [24]. Both individuals with or without neurological disorders were equally likely to consider PMBD, giving relevance to the possibility of being potential donors and, more importantly, showing that further research about different scenarios for PMBD is advisable [25].

Lastly, PMBD provided by specific subgroups of the general population could be of a greater value. Twins samples, for instance, allow effective models of investigation, able to disentangle the contributions of genetic predisposition (‘nature’) and environmental exposures (‘nurture’) to the expression of many complex traits such as neurodegenerative disorders, considering that twins have been shown not to differ from the general population, particularly with respect to social [26] and behavioral [27] characteristics.

The present study is part of a large peer-reviewed Italian project coordinated by the University of Milan: “*Role of LSD1 in Aging-Dependent epigenetic drift leading to FrAilty-associated mood disoRders (RADAR)*”. Among the project aims there was a sensitization programme to enhance awareness among citizens towards PMBD for research in the field of Frailty [28] and Mental Health.

The aim of this study was to explore, among healthy potential brain donors and/or donors’ relatives, attitude and awareness about PMBD, investigating: (i) knowledge regarding PMBD and its procedures, and possible misconceptions; (ii) opinions, beliefs and concerns elicited by PMBD; (iii) initiatives useful to promote PMBD among citizens in the framework of the ethical requirements for research conduct and organs’ procurement.

## Methods

The study adopted a qualitative methodology, carried out with focus group (FG), suitable for exploring data on attitudes and beliefs on emerging themes and scenarios [29]. Considering the sensitivity of the subject addressed and a general lack of knowledge about it, face-to-face FG discussions with one moderator and one observer were considered the best way to identify and resolve misconceptions.

A directed approach to content analysis was adopted to explore and extend understanding of the phenomenon under study [30]. We also grouped and incorporate emotional interactions into the analysis.

Study design was defined by VT and MAS. VT (principal investigator) and CC (co-investigator) developed the interview guide (see Additional file 1) based on the literature as a list of open questions in accordance with the objectives of the FGs, together with a set of basic rules for a productive discussion. The interview guide was tested in a pilot FG held in Rome, questions were checked for capability to stimulate discussion on each topic addressed by the interview.

The outline of a proactive moderation and observation during the sessions was able to provide answers, at the end of each FG, to several questions posed by the participants. A convenient sampling of twins and, at the second stage, a purposeful sampling of their non-twin contacts (i.e. friends, relatives and/or colleagues) was designed. The sampling was aimed at: 1) covering the main macro-areas at national level, given the hypothesis that, for such a sensitive and difficult topic, a great variety of cultural, historical and social background might yeld different opinions, concerns, beliefs; 2) recruiting as many potential donors/donors’ relatives as possible among healthy individuals in the Country, given the experience of a scarce compliance during the recruitment for the pilot FG. The recruitment was, therefore, importantly facilitated by the Italian National Twin Registry (ITR), a research infrastructure of the Italian Public Health Institute (ISS) [31]. Indeed, twin participants helped to recruit the great majority of non-twin individuals. Each twin was an index-subject who captured a non-twin friend/acquaintance or colleague, possibly of the same sex and similar age. In order to avoid a possible bias involving a well-known helpful and trustworthy attitude towards scientific research of twin subjects [32], FGs were conducted separately for twin and non-twin individuals.

FGs were set up in four cities representing all geographic macro-areas of Italy: Milan and Turin in the North, Rome in the Centre, Naples in the South. A total of 12 FGs were performed, stratified according to twins and non-twins status.

### Recruitment

AA, MS and SL conducted the recruitment. A list of potential twin participants, both males and females, aged 50 yrs and over, resident in Italy, enrolled in the ITR, was extracted from the ITR database.

Twins were first asked for participation by email, and progressively enrolled. Those who decided to participate were invited to take part into one FG discussion according to their place of residence. No detailed information regarding PMBD significance and procedures were given during the contact by phone, to avoid a possible selection bias regarding potential participants’ previous knowledge about the topic or undue potential emotional impact on the enrolment effectiveness.

At the beginning of 2019, the enrolment in some areas needed an improvement. The ITR Facebook page was then used and the FGs to be held in Milan and Naples were advertised with dedicated posts.

### Ethical issues and personal data protection procedures

The ISS Ethics Board approved the study on the 6th of June 2018 (Prot. N. PRE.BIO.CE.20580).

Participation to the FGs, the use of audio recording and questionnaires were subject to informed consent procedures designed according to the ethics requirements for research with human beings addressed by the Helsinki declaration [3]. Before the FGs, procedures were also established to let respondents have access to confidentiality and personal data treatment study procedures.

At the beginning of each FG session, a detailed information note was distributed and written consent were provided by participants. Personal data were pseudonymised and recorded separately from audios and questionnaires, according to the EU Regulation n. 679/2016 (GDPR), to guarantee efficient data quality control and to provide participants with follow up information. FGs audio recordings were kept securely, names and other identifiers were redacted from transcripts and debriefing notes. Only aggregated results, anonymous by definition, were presented in the final report of the Project as well as in the present study.

Moreover, to meet an ethical commitment towards citizens who contributed to the realization of the research, participants were provided with a summary report of the overall study and its general findings as feedback on June 2021, by email. The report was specifically designed to be exploitable by lay people, taking into account communication issues and scientific/technical language adaptation.

Furthermore, to enhance participation to the FGs, a final lottery was announced to potential participants. It was conducted at the end of each FG to reward winners (two for each FG) with museum or art exhibition subscriptions. All participants received a USB pen drive as a gift.

### Data collection

The FGs took place from June 2018 to February 2019. A self-administered socio-demographic questionnaire was distributed before FG sessions started, including information about: age, education level, offspring, spirituality and religion. At the beginning of the FGs, participants were thoroughly provided with information about the procedures and the objectives of the study, and at the end of each FG, they received a brochure on brain banking for research. An experienced researcher facilitated the focus groups (CC) in the presence of an observer (VT) who took notes that reflected the dynamics of each group, characteristics of the group conversation (such as active participation, signs of emotion, non-verbal communication). During the session, the observer controlled that all topics were covered and took note of participants’ questions, replying to them at the end of each FG.

During discussion, participants were solicited to share any concerns, questions or opinions associated with PMBD, favouring of an informal environment that made participants feel comfortable in expressing their feelings and concerns. Each FG lasted 1.5–2 h, and was audiotaped; the recordings were transcribed by SL and checked for possible errors and/or misunderstandings by CC and VT.

### Data analysis

A directed content analysis was used to derive themes and sub-themes from data. According to the study design, themes and sub-themes were developed both deductively, based on the analysis of previous literature and, inductively, to be open to further themes and interpretative categories that the discussion let emerge. The choice of maintaining both approaches was made to enhance all the content provided by the gathered data [33] and gain knowledge on potential issues around a delicate topic, not yet completely explored, such as that of PMBD. FGs transcriptions were kept stratified for twin and non-twin participants. CC and VT developed a preliminary coding framework that was applied independently by the two researchers to the whole transcripts to code and classify the data into main themes and related sub-themes. According to the inductive approach, the coding was left open to possible changes as the analysis progressed and “unique” perspectives expressed by an only participant on key issues were also taken into account. The overall transcripts analysis was corroborated by interactions analysis and a couple of emotional interaction categories were identified.

GG and IU revised the codes independently and disparities in the coding of significant patterns of meaning were detected and discussed in three face-to-face meetings by the four researchers (CC, VT, GG and IU), until agreement was reached on discrepancies. Finally, all themes and sub-themes were revised, redundancy was solved and a final interpretation and systematization of results was achieved.

Afterwards, SL chose the most appropriate original verbatim from the FGs transcripts to describe the findings, and IU revised them.

Data management and coding was supported by the use of the software NVivo [34].

A 32-item checklist for the study procedures [35] can be found in Additional file 2.

## Results

Seventy twins and 33 non-twin individuals, between 49 and 91 years, participated in the 12 FGs (2 participants dropped out, 1 in Rome and 1 in Naples. No particular reasons were provided by them). Sixty per cent were females. The percentage of individuals with a university degree ranged from 40 to 100%.

Table 1 reports socio-demographic characteristics of the sub-samples for each FG, and percentages of different opinions about the importance of religion and spirituality in participants’ life.

**Table 1:** The Focus Groups plan; socio-demographic characteristics of participants and reported importance of religion and spirituality

City	Participants	Women	Men	Age Range	University degree	Offspring ^§^	Importance of Religion			Importance of Spirituality		
	N.	N.	N.	Yrs.	%	%	Yes %	No %	I don’t know %	Yes %	No %	I don’t know %
FG TWINS												
Rome 1	16	8	8	50–69	56	69	25	69	6	44	50	6
Rome 2	8	6	2	60–77	50	87	25	50	25	50	25	25
Milan 1	9	6	3	49–72	44	78	67	11	22	100	---	---
Naples 1	10	5	5	51–69	40	70	50	40	10	60	20	20
Naples 2	4	2	2	50–66	50	75	25	25	50	50	25	25
Turin 1 ^	11	8	3	51–90	45	54	55	36		91		
Turin 2	12	5	7	54–75	42	58	58	17	25	67	16.5	16.5
	**70**	**40**	**30**									
FG NON-TWINS												
Rome 3 ^§§^	4	3	1	53–68	75	75	--	--	--	--	--	--
Rome 4^^^^	10	8	2	50–85	60	80			10	70	20	10
Milan 2	4	4	---	50–59	100	75	50	50	---	75	25	---
Naples 3^^^^^	6	3	3	55–69	100	83	33	50		50	17	
Turin 3	9	4	5	52–91	44	78	56	22	22	56	33	11
	**33**	**22**	**11**									
Tot. 12 FG	**103**	**62**	**41**									

§ % of participants in the FG with at least 1 child

§§ Pilot FG in which the items regarding the importance of religion and spirituality were not administered

^ 1 Missing response for Religion and Spirituality importance

^^ 9 Missing responses for Religion importance

^^^ 1 Missing response for Religion importance and 2 Missing responses for Spirituality importance

The following topics and related themes were identified, corroborated by frequent expressions of sincerity and support in the face of the participants’ statements, in particular for what concerned the “role of the family” in PMBD. Moreover, group interactions showed also avoidant statements and attitudes that took shape during the FG discussions in relation to the “brain and identity” theme and “the thought of death” sub-theme (Table 2).

**Table 2:** Themes and Sub-themes derived from the FGs analysis and related key quotes

Themes	Sub-Themes	Verbatim
**KNOWLEDGE ABOUT PMBD**	**TARGET DISEASES**	*“I think… to study increasing diseases such as Alzheimer’s, Parkinson’s, degenerative brain diseases.”* *“This research must be supported because diseases of the brain are particularly excruciating, they keep the body alive and deprive you of identity.”*
	**SCIENTIFIC PROGRESS**	*“The motivation is to contribute to the scientific advancement, to knowledge, to improve quality of life.”* *“Basically, to increase research, to facilitate it and help scientific progress, I think.”*
	**ORGAN DONATION for TRANSPLANT vs. PMBD**	*“I don’t see any difference at this point, one can be used to save a person and the other to save, let’s say in perspective, in the future, with the research.”* *“In my opinion, you can say ‘[I donate] my heart, liver, etc. because you know you are going to save a life anyway. The brain saves nothing in this case.”*
**INFORMATION NEEDS**	**BRAIN DEATH ASCERTAINMENT**	*“Usually, ‘brain death’ is indicated as the end, but I don’t know whether this reported ‘brain death’ is the actual brain death.”* *“Does organ donation take place in a coma defined as irreversible or with brain death?”*
	**SURGERY AND BRAIN PRESERVATION**	*“Having to take a piece of brain tissue, it is of no use if it is taken too late, I do not know technically…”* *“I think that brain tissue can be analyzed but at the same time without deterioration…. I guess, because otherwise it would bias all the results, but this is my hypothesis”*
	**DONOR’S PROFILE**	*“I knew that organs could be donated but a part of me said ‘oh yeah, but you have to be healthy because no one will take your (e.g.) ‘heart’ if you die at 80!… so maybe, no one will be able to benefit because they are no longer suitable.”* *“Actually, why not donate this [brain] as well if it can be useful, I agree, but I don’t know how useful it can be if you donate at a late age.”*
**IMAGINARY**	**BRAIN USED FOR TRANSPLANT**	*“But to donate one’s brain and transplant it into another person who may be sick… I don’t agree, everyone is born and must die with his own brain.”* *“The important thing is that my tissue, […] should be donated only to cure a certain disease. When, on the other hand, you take brain tissue … and the person who receives it has part of my brain, knows part of my life, remembers my past, [I would not agree]”.*
	**BRAIN REMOVAL DURING LIFE**	*“I have a question, even though it is certainly due to my ignorance: is it possible to think about harvesting, obviously in absolutely tiny parts, the brain tissue of living people?”*
**BRAIN AND IDENTITY**	**BRAIN IS ONE’S MIND/THOUGHTS/SELF**	*“Not the brain! The brain is us, our deepest, most refined identity, etc. …do we really have to talk about giving a piece of brain? The brain is me.”* *“One places one’s soul in the brain, one places himself, one’s identity etc. in it, so it is even more important I think than the heart itself.”*
**SPIRITUALITY and RELIGION**	**INTEGRITY OF THE BODY AFTER DEATH**	*“…there may be many people who do not adhere and who for religious belief demand that the body remains intact for burial.”* *“I don’t know, does donating brain tissue mean keeping one’s body intact? I don’t know… “*
	**WHAT HAPPENS AFTER DEATH?**	*“In my opinion, the issue here is this: what happens after death? This is the question; it is a core question in my opinion.”*
	**PROGRESSIVE RELIGIOUS VIEW**	*“Cremation is now accepted, so on a religious level there is no longer [problems]”* *“From a religious perspective, [there is no problem] because certainly it is not in the brain that our soul resides.”*
**ATTITUDE TOWARDS PMBD**	**PMBD AS A GIFT**	*“One can also say ‘I do it for others’, so it is a gift of love, if you like - donating organs or donating anything else […] is an act of love. Whether it is for a person to live or for research.”* *“Spreading the culture of serving our neighbour, when we are gone, in some way contributing to the improvement of the future life of our descendants.”*
	**UTILITARIAN VISION**	*“… we benefit every day from a lot of things that come from the past, from others who preceded us […] and if we are called upon to contribute to research […] that will be useful even if we don’t even know in what exact way… to some extent we have to do it…. that’s the attitude we should all have, someway”.*
	**SUPPORTIVE ATTITUDE**	*“[Donating brain tissue] marks you out as a person who is, however, naturally helpful, naturally supportive. The motivation is to have general solidarity.”* *“I would define the motivation [for donation] like this: knowing that the last act of my life is to do a service to science, in my opinion is an absolutely positive thing.”*
	**BRAIN DECAY vs. BRAIN UTILIZATION**	*“Once that my lifecycle is over, my brain is no longer useful, how can I make it still useful? Leaving something […] can serve a good cause even afterwards.”* *“We’re talking specifically about the brain but I think this applies to all organs, instead of throwing it away, it can be of help for something.”*
**ISSUES ELICITING NEGATIVE EMOTIONS**	**BRAIN DEATH “UNCERTAINTY"**	*“Let’s be honest, one thinks about dying and the fear, at least my personal fear, is to have an apparent death, […] this idea terrifies me, it scares me.”* *“It’s definitely an emotionally challenging moment, I mean […] for the brain death assessment it happened to me to hear the relatives say: ‘what if the equipment doesn’t work properly?’*
	**CRUELTY OF SURGERY**	*“To think of having [one’s head] drilled, […] this would have immediately made anyone say: I refuse!”* *“It’s true […]if you take this decision they will really do it! For real, they chop you up and distribute you! That made a bit of an impression on me.”*
	**THE THOUGHT OF DEATH**	*“Talking about death is like looking at the sun for too long, you can’t stare at it, you turn your face away - that’s the meaning. You can’t stare at the sun for too long. Because it’s our culture, one avoids that talk.”* *“The brain has a different impact on imagination […]The moment when the relatives have to face the question of donation, they say ‘no, please, I don’t want to hear about it, because… I don’t want to think about death’.”*
	**MENTAL DISEASE REJECTION**	*“Brain is a part of the body that has always been considered special […] everyone has a certain degree of reluctance to think or speak about mental diseases […] and to think about PMBD recalls mental diseases.”*
**FAMILY MEMBERS’ ATTITUDE**	**CENTRALITY OF THE DYING PERSON’S CHOICE**	*“If my family member [dying] had voiced his will, I would respect this will, absolutely I would never go beyond, even if I did not agree I would never go beyond his choice.”* *“This situation is a complicated matter: there is no explicit consent and no explicit dissent, I have to take responsibility for another person […] I don’t know what to say.”*
	**STRESS, GRIEF and CONCERNS**	*“When my mum died […] I was so disheartened, so sad, so stressed, I can even say so very angry […] and so I definitely would have said ‘no’!”* *“I think that when you have a loved one dying, anyone who approaches you and tries to say anything other than ‘I’ll cure her/him’, you just kick them out, in a rude way or politely, but anyway, you kick them out.”*
	**OPINIONS ON THE AGREEMENT TO CONSENT**	*“I think that all the family members should come to an agreement, when my mum died one of them could say OK, but what if the others said no?”* *“I am in favor [of PMBD], also because I plan to give my consent to cremation so there is no problem. The only thing is that I would like to discuss it with my family, because I think they would have a major problem with it, certainly not me.”*
	**“ONLY FOR A CLOSE RELATIVE?”**	*“In my opinion, the decision can be made only by a partner, secondarily by parents/brothers/children.”* *“This issue is difficult if your own child is the dying person. I do admire those mums who consent. What courage!”*
	**MISTRUST**	*“So, I might give my consent, but tomorrow whoever is in charge of implementing my will could say ‘no, I do not approve’.”* *“If I have this will, if I want to make this life choice, somehow I leave it in writing, I declare it somewhere, I register it otherwise neither my daughter nor my wife nor the doctor can decide it for me, this is my point of view.”*
	**EXPERIENCE of a NEURODEGENERATIVE DISEASE**	*“My dad in the end had Alzheimer’s, I think for what concerns the brain tissue if they had asked me […] I would have been more inclined [to give consent] to promote the research.”* *“My experience influences me, if you talk to me about Parkinson’s you have my full attention, .my experience in these matters has much impact on me, that makes a difference.”*
**TRUST**	**RESEARCH SOUNDNESS**	*“On brain tissue, in my opinion, there are fears and qualms because it is seen as a different organ from the others. […] One should check the aims of such research.”* *“Certainly, the aims must be made very clear!”*
	**RESEARCH TRANSPARENCY**	*“I’m speaking in general, it may well be that in order to reach certain goals one can also do things that are [not perfectly clear].”* *“As it has happened so many times in research, it’s not that there haven’t been cases […] Mine is just a question.”*
	**INSTITUTION REPUTATION**	*“Since this request came from [an accredited institution], I didn’t worry about the question, but considering that today there are all these fake news […] if there is no information […]”*
	**ETHICAL ASPECTS**	*“These are processes difficult to control, as the history has taught us how research can evolve up to very dangerous frontiers.”* *“An obstacle could also be if you think that the study is not for everyone’s benefit, that the study is done to be then manipulated.”*

### Knowledge and information needs

In general, participants had scarse knowledge regarding PMBD and they showed a need to better understand its aims and procedures.

When solicited to think about the aim of PMBD, many hypotheses were made. They mainly addressed the research on brain structure and functions; genetic investigations; neuro-degenerative diseases investigations; studies on the relationship between brain anatomy and functioning and pathological manifestations. A general “scientific progress aim” was mentioned. This last theme emerged mostly among twins.

It is noteworthy a recurrent mix-up, observed through all the FGs, between organs donation for transplant (OD) and PMBD.

Participants asked recurrent questions highlighting basic doubts about several aspects of both OD and PMBD. Mainly, queries focused on surgery procedures, organisational aspects of brain procurement and tissue storage time. Brain procurement was often imagined as involving a portion of the brain, only very few individuals thought the donation might regard the whole brain.

The need to receive detailed information about these topics and to understand how brain death is ascertained was apparent. Further participants’ interests focused on the aims of the research requiring cerebral tissues and on the need to receive feedback on possible research findings.

A recurrent question concerned the “donor profile” for PMBD, this might be explained with an underlying (sometimes also declared) need to know whether the brain for research purposes should be from a healthy donor or a diseased one. Moreover, participants variously asked whether the brain from a deceased person could be “*useful*” any longer, underlying a tendency to make confusion between OD and PMBD for research purposes. This slippery slope reasoning was verified in all FGs.

### Imaginary and conceptualization of the “brain”

Particularly in the FGs in Rome, many participants imagined that the donated brain might be utilized for transplantation in another individual. A misunderstanding regarding the procurement of the brain not post-mortem but during life often emerged as well.

Moreover, the brain was often conceptualized as the Ego, the natural seat of the Self; many declared that its importance and value was not comparable with other parts of the human body. Consequently, arising emotions and fear for its loss emerged during discussions. Several participants expressed the idea that donating the brain is like donating one’s own thoughts, one’s own mind. A secondary opinion by some participants focused on a widespread reluctance to undergo mental health therapies: “*we do not have a culture of this things”* [NdA: of treating brain pathologies] (FG Naples), that might be explainable by the fact that “*the brain is not the heart or the liver…*” (FG Rome).

### Religion, spirituality and solidarity

Religious precepts such as the inviolability of the integrity of the human body was addressed by a few participants, and uncertainty about what may happen after death emerged: *“one never knows what may happen after death*”. Some individuals were worried that the loss of such an essential part of the body cannot be conjugated with life after death.

A deriving conflict between OD and PMBD and burial or cremation procedures was also highlighted. Discussions around a common moral conflict occurred in various FGs that can be synthesized as follows: “*should I donate to help others or shouldn’t I for my religious faith and the consequent respect of the integrity of the body*?”. At the same time, the opinion that religion (i.e. Catholicism) has now developed broad-minded positions against therapeutic obstinacy, and other progressive ideas was addressed, making it easier the adoption of more favourable positions towards PMBD and OD.

Conversely, a secular view was detected across all the FGs when informants expressed outright pro-PMBD support. This view mainly addressed the idea that after death “*nothing really has value anymore*”. Moreover, both OD and PMBD were seen as a civil duty: *“a part of myself can be useful and I can embrace a civil view of donation”*. Other interpretative frameworks were: PMBD “*gives significance to life*” or represents a “*gift*” or “*an actual contributing engagement*”.

### Issues eliciting negative emotions

Difficulty to cope with the issue of “death” and “disease” was prevailing across all the FGs. Some participants underlined that events such as “death” or “disease” were psychologically better managed by people in the past.

The ascertainment of brain death was a main concern, around which many worries and fear (sometimes anxiousness) emerged.

Many individuals envisaged that they might experience negative emotions if “*forced*” to think about the removal of the brain as this can evoke images of “*cruelty*” of the surgical procedure. In particular, in Turin the need to “*elaborate*” and “*accept*” in advance the idea of the brain explant was clearly explained; it was also deemed that, in itself, the surgery has a certain undisputable degree of violence.

A feeling of reluctancy was reported when coping with mental illness while facing PMBD. A few individuals in Milan underlined the usefulness of meetings such as FGs to speak about mental diseases, they felt they might facilitate acceptance.

Finally, the donation of a portion instead of the whole brain was considered far less worrying and emotionally more acceptable by the most participants in all the FGs.

### The role of family

A higher attention concerning the role of “family” was recorded during the FGs in Naples, compared to the other FGs, even if “family” was found to be a key issue in the process of decision making for PMBD across all the FGs, sometimes controversially. For this reason we deemed that the “role of family” deserved a thoroughful examination.

In particular, the decision taken by people during their conscious life was considered, almost unanimously, essential. In general, only if the dying person had not been clear about his/her will for PMBD, their next of keen were allowed to give a consent for it.

Other recurrent comments underlined the heavy responsibility of family members for the decision to be taken and the difficulties they can meet when the dying person is a loved one. In Naples, it emerged that family members’ bereavement grief is a major obstacle for a relative to choose PMBD. Moreover, a few participants who declared their willingness to donate their brain, felt that it was necessary to inform their general practitioner, or the hospital staff as they thought that only a clear official procedure would really guarantee their will.

The duty to take into account the opinion of each family member and to respect their sensitiveness when making the choice for PMBD was also emphasised. Few individuals expressed their preference to leave the choice to the children at the time of their death.

In Turin, one participant showed a real concern for his family members, believing that they would inevitably face moral, psychological and emotional difficulties in making the decision to consent to PMBD for him.

In Rome, the opinion that consent for PMBD can be given only by close relatives (spouses, children, brothers and sisters) was clearly expressed; a few participants directly stated that they would give consent to PMBD if they had a dying relative. A solid reason to opt for PMBD was the presence of a neurodegenerative or neurological disease within the family, which was seen as a strong facilitating factor.

### Attitudes towards organ donation and PMBD

From a cultural and social perspective, a potentially positive attitude towards PMBD was highlighted when participants referred to the comparability of OD and PMBD in terms of “life-saving” activities, the former to directly save another life, the latter to improve research on specific diseases that might help to save lives in the future. Another speculative view focused on the chance of “prolonging life” and giving meaning to a dying persons’ life through PMBD. Someone explored the subject and came to a practical consideration regarding the usefulness of using the brain deliberately against its inevitable deterioration.

Various opinions can be viewed within an “altruism and solidarity” framework. In this category, there were ideas of a general solidarity that donors can have towards society for biomedical progress and, sometimes, for a sense of social responsibility towards other humans. In the same area, a sort of “utilitarian” attitude also emerged, with some participants talking about a costs-benefits balance between the benefits gained from previous biomedical research advancements deployed in our times, and the act of PMBD today for medical improvements in the future.

### Trust

Trust was another key element raised several times during discussion. It involved themes such as reliability of research, of the institutions/investigators in terms of their reputation and, more generally, of research transparency. Many individuals highlighted the threat of unjustified experiments and/or manipulations, and the risk of an undue brain tissue removal before the ascertainment of the brain death. Potential unjustified commercial deployment of the biological specimens was also mentioned. Conversely, a few people reported a high level of acceptance, and a relaxed attitude: “*… Once I have donated, I forget it”.*

### How to promote PMBD

Participants’ attention frequently focused on families of people affected by mental and neurodegenerative diseases as the most suitable “micro environment” to be comfortably informed about PMBD. Many participants thought that only an open dialogue between health professionals and family members could raise awareness on the topic.

Education emerged as crucial. According to most of the participants, school and communitarian environments might play an important role in educating young people to responsible solidarity.

Regarding the most helpful topics to be propedeutically developed for ehancing PMBD, “mental health and neurodegenerative diseases**”** were largely addressed, especially those affecting elderly people. Furthermore, “aim and objectives of the research that makes use of the brain tissue” should also be highlighted to promote PMBD and, in particular, both distinction and comparability of PMBD and OD for therapeutic purposes should be well disseminated.

Participants identified health professionals and general practitioners as possible “actors” to be involved in raising awareness and informing about PMBD.

Specific tools to formalize individual informed consent for PMBD were mentioned, such as the living will, or a registration in a personal card, similarly to the OD acceptance on the Identity Card, legally required in Italy. Finally, a large number of individuals thought there was a need for new standards to regulate PMBD.

Themes and subthemes derived from the analysis are reported with a few key quotes from participants in Table 2.

## Discussion

The results showed that PMBD was a new topic for the great majority of participants. Despite a more frequently reported general enthusiasm for scientific research detected during the FGs of twin subjects, no evident differences between twin and non-twin groups emerged regarding knowledge, attitudes and concerns about PMBD.

Participants were often inclined to express themselves in terms of what “others” (e.g. their own relatives) or “society” as a whole might think about the issue of PMBD. The complexity of the potential role of family members, with multiple and often conflicting opinions, was striking and it is a distinctive finding of our study.

Indeed, while several previous studies detected single themes regarding the role of family [10, 11, 18], our results showed a great variety of themes concerning role, influence and opinions of family members in the process towards PMBD. All of them together confirm the importance of family as an emotional and cultural environment playing a key role for PMBD. The importance of a “family-centered” decision-making process was noticeable, in particular in the FGs conducted in the South (Naples). Given cultural and historical differences among Italian areas, this could be due to the fact that family influences are more strongly rooted in specific cultural and social environments and play a crucial role in sensitive decisions [36].

Family sub-themes were sometimes conflicting, ranging from the appreciation of family members’ involvement in the decision-making process, to a resentment or a concern that family members might not respect the will of their decesead loved one. A potentially highly interfering family role towards PMBD was already known [10, 11, 37], and our study supports this idea. Strong feelings and concerns were envisaged as affecting family members: high sense of responsibility, much grief over the loss of a loved one, high level of uncertainty about family members respect of their loved one’s will. These concerns were complemented by different factors; an hesitation caused by the lack of a clear expression of the dying person’s wishes during life; a sort of empathic concern experienced by participants, in perspective, for a possible refusal of PMBD by their own family members; or an undeniable worry for the intensity of grief and stress their family members might feel when called to give consent. This last dynamic was well underlined in previous research [25].

Compared to healthy individuals, motivations leading to PMBD are more easily detectable among patients with neurological or neurodegenerative diseases and their families, as the disease is a central factor in shaping willingness to donate [25, 37]. Yet, the need to better understand the dynamics influencing PMBD in healthy populations has been well recognized [10]. Moreover, a recent systematic review [38] showed that conceptual understandings, family situation and personal experiences were powerful features to inform decision for PMBD. The relevance of these same factors are, directly or indirectly, detected in our study. Actually, for what regards personal experience, it resulted often out of reach, and opinions were driven more by an intellectual approach. At the same time, the great amount of information needs expressed by participants about aims, procedures and mechanism of PMBD addresses, indirectly, the importance of health literacy to facilitate the process of its acceptance [10, 11, 37]. Our study recorded, in fact, poor knowledge among individuals, as well as a recurring misunderstanding between OD and PMBD consistent with earlier research [10, 11, 37, 39]. This misunderstanding, which often lurked in the background of the discussion, was highly linked to imaginary and fantasy, together with other main factors such as the identification of the “brain” with the “Self”, several negative emotions elicited by the thought of “death” and, a high degree of uncertainty about the definition and ascertainment of brain death.

Indeed, the ascertainment of brain death emerges as a potentially hindering factor for PMBD, and following this issue, many individuals landed on an emotional non-acceptance of “death” and “illness” that are likely to play a major role in personal decision-making. They declared a sense of ambiguity about brain death ascertainment and an underlying hope that a dying individual *“might recover from it*”. However, there is contrasting evidence on this issue: even if PMBD rates are higher among those with higher knowledge regarding brain death, an accurate knowledge about brain death does not seem to be a driving force of the willingness to donate [40]. Other research has, in fact, shown only a moderate relationship between participants’ knowledge of life-prolonging medical procedures and willingness to PMBD [16]. Whatever the case, a regulatory debate about the definition of brain death is still ongoing [41], and empirical research shows significant lack of acceptance of brain death ascertainment among individuals [42]. It is, therefore, undeniable that the debate may raise concerns among people, and our findings suggest that the perceived lack of reassurance about brain death ascertainment should be further investigated among potential donors. To overcome this frequent misunderstanding related to the emotional reaction in front of an issue such as that of brain death, it is important to clearly address the fact that only people who have suffered cardiac death are eligible as brain donors, and it is also important to clarify that cardiac death is unambiguous and faster to be ascertained. The real problem to face is that of the post-mortem interval to explant, which should be as short as possible (less than 24–30 h) [14].

Central was also an underlying role of religion and spirituality in framing events and decisions concerning death and PMBD, it emerged in our study as in previous research. A few studies, in fact, identified a positive contribution of religious beliefs [11, 21], others observed mainly an impeding role deriving from religious concerns [10, 25, 37]. We noted contrasting contributions: an intention of preserving one’s integrity, with indirect rejection of “body disfigurement” as a consequence of surgical removal of the brain, but also a sensitive and altruistic attitude towards PMBD and its positive impact on others, linked to religious precepts. This altruistic attitude was sometimes justified by participants as a result of an increasingly progressive contemporary view of religion.

“*A part of me, of my body, may be useful, therefore I may help others this way*” is an expression that summarizes an emerging general solidarity; many individuals spoke about PMBD as a “*good act*” to help others through potential research advancements or in terms of “*gift*”. A few opinions were formulated, from a utilitarian perspective, showing the importance of a trade-off between benefits and costs. In this perspective, one can take advantage from the biomedical findings to which other people contributed in the past, and, correspondingly, one has got a chance, sometimes considered a duty, to give a contribution for the generations to come.

Overall, the role of solidarity, framed by utilitarianism, religion or spirituality had a clear place in the discussion in favor of PMBD.

Lastly, the topic of a societal “trust” towards research and its actors emerged as well. Concerns about possible inappropriate procedures or unclear research aims, and subsequent lack of trust towards researchers and physicians, were already found as pivotal drivers in shaping the behaviour of healthy individuals towards PMBD [39].

From a legal perspective, it is also important to point out that in Italy steps forwards were recently made to highlight as central the role of one’s deliberate choice and free will to donate one’s body for research purposes [43]. However, the recent law issued in 2020 [44] doesn’t address PMBD and its specific procedures. Therefore, an important legislative gap still exists.

For what concerns limitations, the choice of the method was crucial to an in-depth investigation of opinions and concerns about a quite an unknown topic such as PMBD. However, the study has the limits of a qualitative research, there is a need to implement quantitative studies on wider samples drawn from the general population, to assess the impact of the several emerged factors in relation to PMBD. Moreover, the study investigates opinions and concerns in a theoretical framework, further research is necessary for detecting reactions on the ground of concrete proposals to join a PMBD programme.

Finally, there was a high rate of refusal of participation in the South of Italy (i.e. islands), and it will be necessary to investigate this phenomenon promoting additional research.

## Conclusions

The complexity of the process leading to PMBD emerged clearly. Our study reinforces the need for public interventions to promote PMBD, as already addressed by various scholars during the last decades [21, 37]. In particular, it will be central to evaluate medical personnel’s ability to communicate the importance of PMBD as well as to address the concerns of potential donors or their families. In this line, a territorial or an institutional framework may be necessary to create a network of highly trained medical professionals (specialists and general practitioners) with a strong empathetic and careful ethical approach, willing to raise awareness on this issue [14].

However, we deem of importance that an inner understanding of the role of the brain as conceptualized among individuals and within society should be encouraged while promoting PMBD outside the clinical context. Moreover, cultural efforts are needed to support a larger societal acceptance of “death” and “disease”, as well as to promote an ethical debate for the best poise between the value of scientific and health care advancements and the inner needs of human beings.

### Electronic supplementary material

Below is the link to the electronic supplementary material.

Supplementary Material 1

## Data Availability

The dataset analysed during the current study is available from the corresponding author on reasonable request.

## Declarations

Participation to the FGs was subject to informed consent procedures designed according to the ethics requirements for research with human beings, addressed by the Helsinki declaration.

## List of abbreviations

PMBD: Post mortem brain Donation
OD: Organ Donation
ISS: Italian National Institute of Health
