# Supplementary material for: Attitude and concerns of healthy individuals regarding post-mortem brain donation. A qualitative study on a nation-wide sample in Italy
Source: BMC Med Ethics. 2023 Nov 27;24:104. doi: 10.1186/s12910-023-00980-3 (PMC10683267; doi:10.1186/s12910-023-00980-3)
Supplement: Supplementary file 1 — Supplementary Material 1 [file 12910_2023_980_MOESM1_ESM.docx]

**Additional file 1**

**Interview Guide**

Note for the FG conduction: answers to possible requests for explanation by participants and other queries will be provided at the end of each FG sessions, the observer will take notes of them. Answers will be given immediately, in case it is useful for the discussion.

**Presentation:** the study, its objectives and the FG procedures are presented to participants (5 min.).

Introducing Conductor and Observer (10 min.).

Warm up questions

**First objective: explore knowledge about post-mortem donation of cerebral tissue**

**by participants** (20 min.).

1. Have you ever heard about pmbd, before now?
2. If you have, in which context?
3. In your opinion, what is pbmd for?

3. How do you imagine the process leading to pbmd?

**Second objective: to explore opinions, emotions, concerns elicited by pmbd** (15 min.).

1. What do you think about and which emotions the topic of pmbd arouse?

2. Do you think there are any differences between pmbd and organ donation for transplant?

Try to explain them.

**Third objective: to explore which information, tools and procedure are considered useful to favour pmbd** (20 min.).

1. In the light of what we have just discussed, do you think there are important information to promote pmbd among people? If yes, what are they?

2 In the light of what we have just discussed, do you think there could be specific tools/actions able to promote pmdb? If yes, what are they?

**Question time** **and responses** (about the Project, its different aims, etc) (15 min.).

**Additional file 2**

**Consolidated criteria for reporting qualitative studies (COREQ): 32-item checklist (Tong et al. 2007)**

| **No.** | **Item** | | **Description** | **Section #** |
| --- | --- | --- | --- | --- |
| **Domain 1: Research team and reflexivity** | | | | |
| Personal characteristics | | | | |
| 1. | Interviewer/facilitator | | *Which author/s conducted the interview or*  *focus group?* CC (conductor) and VT (observer) | Research Design and Methods/Data collection |
| 2. | Credentials | | *What were the researcher's credentials?* CC is PhyD in Health Psychology and MSc in Psychology  VT is a Sociologist and MA in Bioethics | Title page |
| 3. | Occupation | | *What was their occupation at the time of the study?* Researchers in Epidemiology, Public Health and Health promotion, at the Italian Institute of Health | / |
| 4. | Gender | | *Was the researcher male or female*? Both females | / |
| 5. | Experience and  training | | *What experience or training did the researcher have?* CC is expert in FG conduction and VT in behavioural research | / |
| Relationship with participants | | | | |
| 6. | Relationship  established | | *Was a relationship established prior to study commencement?* Twin individuals were already enrolled in the Italian Twin Registry in previous years, they had given a consent to be contacted to participate in future studies, but no relationship had already been established, before recruitment, regarding this specific research objective. Non-Twin individuals had no prior relationship with Interviewers before the FGs. | Research Design and Methods |
| 7. | Participant knowledge of the interviewer | | *What did the participants know about the researcher? E.g. Personal goals, reasons for doing the research*  Interviewers were introduced to participants just before the FGs started | / |
| 8. | Interviewer characteristics | | *What characteristics were reported about the interviewer/facilitator? E.g. Bias, assumptions, reasons and interests in the research topic*  Participants were informed about the role of the interviewers in the ISS organization; their credentials, infos regarding the funding of the whole Project. | / |
| **Domain 2: Study design** | | | | |
| Theoretical framework | | | | |
| 9. | Methodological orientation and theory | | *What methodological orientation was stated to underpin the study? E.g. grounded theory, discourse analysis, ethnography, phenomenology, content analysis* Directed content analysis | Research Design and Methods/Data analysis |
| Participant selection | | | | |
| 10. | Sampling | | *How were participants selected? E.g. purposive, convenience, consecutive, snowball*  Convenience and, at a second stage, purposive, with one-indexed twin-subject recruiting another subject from the general population matched for sex and age (twin vs non twin individuals) according to the evidence that twins are more interested in participating and donating for research, FGs were stratified for twins and non-twins. | Research Design and Methods |
| 11. | Method of approach | | *How were participants approached? E.g. face- to-face, telephone, mail, email*  Email and, in a second step, telephone calls | Research Design and Methods/Recruitment |
| 12. | Sample size | | *How many participants were in the study*?  103 ( 41 males and 62 females) | Results |
| 13. | Non-participation | | *How many people refused to participate or*  *dropped out? What were the reasons for this?*  2 participants dropped out in Rome and Naples FGs. No particular reasons were provided by them. | Results |
| Setting | | | | |
| 14. | Setting of data  collection | | *Where was the data collected? E.g. home, clinic, workplace*  Iss in Rome; GPs Order location in Turin; University of Milan; Public Hospital in Naples. | Research Design and Methods |
| 15. | Presence of non-  participants | | *Was anyone else present besides the*  *participants and researchers?*  One son of a very old participant in Turin FG was present | / |
| 16. | Description of sample | | *What are the important characteristics of the sample? E.g. demographic data, date* See Table n.1 | Results/Table 1 |
| Data collection | | | | |
| 17. | Interview guide | *Were questions, prompts, guides provided by the authors?* Yes  *Was it pilot tested?* Yes.  There was a pilot FG in Rome | | Research Design and Methods |
| 18. | Repeat interviews | *Were repeat interviews carried out? If yes, how many? N.A.* | | / |
| 19. | Audio/visual recording | *Did the research use audio or* *visual recording to collect the data?*  Yes, audio recorded | | Research Design and Methods/Ethical issues and personal data protection procedures |
| 20. | Field notes | *Were field notes made during and/or after the interview or focus group?*  Yes, during the FGs from the observer | | Research Design and Methods/Data collection |
| 21. | Duration | *What was the duration of the interviews or*  *focus group?*  1h and half up to 2 hs. | | Research Design and Methods/Data collection |
| 22. | Data saturation | *Was data saturation discussed?* N.A. | | / |
| 23. | Transcripts returned | *Were transcripts returned to participants for*  *comment and/or correction*? No | | / |
| **Domain 3: analysis and findings** | | | | |
| Data analysis | | | | |
| 24. | Number of data  coders | *How many data coders coded the data?* Two data coders and 2 reviewers. | | Research Design and Methods/Data analysis |
| 25. | Description of the  coding tree | *Did authors provide a description of the coding tree?* Yes. | | Results/Table 2 |
| 26. | Derivation of themes | *Were themes identified in advance or derived* *from the data?* Both | | Research Design and Methods/Data analysis |
| 27. | Software | *What software, if applicable, was used to*  *manage the data?* NVivo (Version 12Pro, QRS International) | | Research Design and Methods/Data analysis |
| 28. | Participant checking | *Did participants provide feedback on the*  *findings?* No, but they were sent a summary report of findings by email in June 2021 and asked to reply whether they had any questions. | | Research Design and Methods/Ethical issues and personal data protection procedures |
| Reporting | | | | |
| 29. | Quotations presented | *Were participant quotations presented to illustrate the themes / findings?* Yes.  *Was each quotation identified? E.g. Participant number* Quotations were not identified | | Results/Table 2 |
| 30. | Data and findings  consistent | *Was there consistency between the data*  *presented and the findings?* Yes | | / |
| 31. | Clarity of major  themes | *Were major themes clearly presented in the*  *findings?* Yes, each theme had a section | | Results |
| 32. | Clarity of minor  themes | *Is there a description of diverse cases or*  *discussion of minor themes*? Yes, important unique opinions were taken into account and minor themes that inductively came out from the FGs | | Research Design and Methods/Data analysis |

Allison Tong et All. Consolidated criteria for reporting qualitative research (COREQ): a 32-item checklist for interviews and focus groups, *International Journal for Quality in Health Care*, Volume 19, Issue 6, December 2007, Pages 349–357, <https://doi.org/10.1093/intqhc/mzm042>
